# Retrieval of the Vacuolar H^+^-ATPase from Phagosomes Revealed by Live Cell Imaging

**DOI:** 10.1371/journal.pone.0008585

**Published:** 2010-01-05

**Authors:** Margaret Clarke, Lucinda Maddera, Ulrike Engel, Günther Gerisch

**Affiliations:** 1 Program in Genetic Models of Disease, Oklahoma Medical Research Foundation, Oklahoma City, Oklahoma, United States of America; 2 Nikon Imaging Center at the University of Heidelberg, Bioquant, Heidelberg, Germany; 3 Max-Planck-Institut für Biochemie, Martinsried, Germany; University of Birmingham, United Kingdom

## Abstract

**Background:**

The vacuolar H+-ATPase, or V-ATPase, is a highly-conserved multi-subunit enzyme that transports protons across membranes at the expense of ATP. The resulting proton gradient serves many essential functions, among them energizing transport of small molecules such as neurotransmitters, and acidifying organelles such as endosomes. The enzyme is not present in the plasma membrane from which a phagosome is formed, but is rapidly delivered by fusion with endosomes that already bear the V-ATPase in their membranes. Similarly, the enzyme is thought to be retrieved from phagosome membranes prior to exocytosis of indigestible material, although that process has not been directly visualized.

**Methodology:**

To monitor trafficking of the V-ATPase in the phagocytic pathway of *Dictyostelium discoideum*, we fed the cells yeast, large particles that maintain their shape during trafficking. To track pH changes, we conjugated the yeast with fluorescein isothiocyanate. Cells were labeled with VatM-GFP, a fluorescently-tagged transmembrane subunit of the V-ATPase, in parallel with stage-specific endosomal markers or in combination with mRFP-tagged cytoskeletal proteins.

**Principal Findings:**

We find that the V-ATPase is commonly retrieved from the phagosome membrane by vesiculation shortly before exocytosis. However, if the cells are kept in confined spaces, a bulky phagosome may be exocytosed prematurely. In this event, a large V-ATPase-rich vacuole coated with actin typically separates from the acidic phagosome shortly before exocytosis. This vacuole is propelled by an actin tail and soon acquires the properties of an early endosome, revealing an unexpected mechanism for rapid recycling of the V-ATPase. Any V-ATPase that reaches the plasma membrane is also promptly retrieved.

**Conclusions/Signficance:**

Thus, live cell microscopy has revealed both a usual route and alternative means of recycling the V-ATPase in the endocytic pathway.

## Introduction

The vacuolar H^+^-ATPase, or V-ATPase, is a multi-subunit enzyme conserved in all eukaryotes that uses the energy from ATP hydrolysis to move protons across membranes. The enzyme is divided into two sectors, each containing multiple subunits. The peripheral V_1_ complex is responsible for ATP hydrolysis, and the membrane-spanning V_0_ complex is responsible for proton translocation. The proton gradient generated by the V-ATPase is used to energize the uptake of small molecules such as neurotransmitters, to regulate extracellular pH, and to acidify the lumen of organelles. Acidification of endosomes and phagosomes by the V-ATPase mediates receptor recycling, activation of lysosomal enzymes, and killing of pathogens. There are several means of regulating V-ATPase activity, including control of V-ATPase subunit expression, intracellular targeting and recycling of V-ATPase-containing vesicles, reversible dissociation of the V_0_ and V_1_ domains, and modulation of the coupling ratio between ATP hydrolysis and proton pumping [1]–[4].

The professional phagocytes of *Dictyostelium discoideum* have proved to be an excellent system for analyzing the V-ATPase in the endocytic pathway [5]–[12]. *Dictyostelium* cells will ingest a wide range of particles including bacteria, yeast, latex beads, glass microspheres, and occasionally other cells. They can serve as hosts for a number of bacterial pathogens that also infect human cells [13], [14]. Many aspects of the endocytic pathway are conserved between *Dictyostelium* and mammalian phagocytes, including mechanisms of particle recognition, the role of the cytoskeleton in uptake, the delivery of digestive enzymes, and membrane retrieval [11], [15], [16]. Here we add the localization of phosphatidylinositol (3)-phosphate, or PI(3)P, to vesicle membranes in *Dictyostelium* cells as a common marker of the early endosomal compartment.

PI(3)P is one of a group of phosphoinositides that specify the identity of compartments along the endocytic pathway by recruiting signaling proteins that carry specific phosphoinositide-binding modules [17], [18], [19]. Studies with mammalian phagocytes have shown that PI(3)P is enriched in the membrane of early phagosomes starting about 60 seconds after the phagosome seals and lasting 8 to 10 minutes [20], [21], identifying the sorting stage of endocytic transit. PI(3)P recruits ligands that contain FYVE domains, zinc-finger domains of ∼70 amino acids present in a number of signaling proteins [18]. The FYVE domain fused in one or two copies to the C-terminus of GFP acts as a probe in living cells for membranes enriched in PI(3)P [22], [23]. We have employed this probe to identify the stage of endosomal transit during which the V-ATPase is added to the phagosome membrane.

Using a fusion of GFP to the large trans-membrane subunit of the V-ATPase (called VatM in *Dictyostelium* and subunit a in mammalian cells), we previously demonstrated that shortly after internalization the V-ATPase is delivered to the membrane of new phagosomes by fusion with acidic endosomes that bear the V-ATPase in their membranes [8]. Recently, a related study in mouse macrophages found that lysosomal V-ATPase is recruited directly to phagosomes via tubular lysosomes and is responsible for phagosome acidification [24], confirming the similarity of the two systems. In their natural environment, *Dictyostelium* cells rely on phagocytosis for the acquisition of food, so the endocytic pathway is a high-throughput system that ends with the exocytosis of indigestible food residues. Although macrophages do not explicitly mimic this behavior, regulated exocytosis from the endocytic pathway occurs for mammalian cells in several contexts such as healing a torn cell surface [25], increasing plasma membrane area during fibroblast spreading [26], and exocytosis of secretory lysosomes by immune cells [27].

Following exocytosis in *Dictyostelium*, late endosome membrane markers, but usually not the V-ATPase, are found in the plasma membrane at the site of exocytosis [8], [28], [29]. Furthermore, VatM protein is maintained at normal levels for days even when VatM mRNA has been reduced to trace levels using antisense methods [30], implying that the enzyme is efficiently reutilized. Taken together, these data argue that the V-ATPase is normally recycled prior to exocytosis. Similarly, intracellular targeting and recycling of V-ATPase containing vesicles are known to be important means of regulating V-ATPase function in mammalian cells, especially osteoclasts and cells involved in controlling acid-base balance [1], [31]. However, these processes have not been visualized in living cells.

In the present study, we have employed VatM-GFP to visualize trafficking of the V-ATPase in the endocytic pathway of living *Dictyostelium* cells, with the primary focus being on retrieval of the enzyme from phagosomes. As particles to be tracked through the pathway we have used living yeast. Yeast cells contain digestible nutrients as do bacteria, but they are larger and their cell wall maintains its shape up to exocytosis. To distinguish the particle surface from the phagosome membrane, we have used a yeast mutant that forms buds but is impaired in division, providing the particle with a characteristic profile. To distinguish acidic and neutral compartments, we conjugated fluorescein isothiocyanate (FITC), a pH-sensitive fluorophore, to the yeast surface.

In live-cell studies of endocytic trafficking, early events are relatively easy to capture because they occur promptly and frequently when a population of phagocytes is presented with appropriate target particles. Thus, we have previously shown that shortly after uptake new bacteria-containing phagosomes are transported along microtubules to the cell center, where they fuse with acidic endosomes (presumptive lysosomes) and become acidified themselves (as detected with neutral red) about five or six minutes after uptake [12]. However, events at the end of the endocytic pathway take place with little synchrony over several hours, necessitating longer observation periods to capture infrequent events and making photobleaching and phototoxicity major obstacles. Only recent advances in imaging techniques [32] have allowed visualization of rapidly-moving vesicles bearing VatM-GFP at high temporal resolution and with minimal photobleaching, permitting images to be captured for up to twenty minutes at 1-second intervals. These time series have provided the first views of retrieval of the V-ATPase and have revealed multiple mechanisms by which retrieval can be accomplished in the phagocytic pathway of *Dictyostelium*.

## Materials and Methods

### *Dictyostelium* strains, cell culture, and vectors

*Dictyostelium discoideum* strain AX2–214 was transformed by electroporation using vectors for the expression of mRFPmars-LimEΔcoil [33] (called ‘mRFP-LimEΔ’ here) or DdmCherry-LimEΔ [34] and either VatM-GFP [8], GFP-MyoB (a gift of Margaret Titus), or GFP-2FYVE. The GFP-2FYVE vector for *Dictyostelium* was constructed as follows. A SmaI-XhoI 2FYVE restriction fragment was excised from the plasmid pEGFPC1-2FYVE [23] and inserted into the SmaI-XhoI sites of pBluescript SK+ (Stratagene). From the resulting plasmid, a 545-bp XhoI-XbaI fragment was excised, then inserted into the *Dictyostelium* expression vector pTX-GFP [35] cut with the same two enzymes.

*D. discoideum* strains were cultivated axenically at 22°C in nutrient medium containing selective agents (G418 and blasticidin) for maintenance of the plasmids. For phagocytosis experiments with living yeast cells, *Saccharomyces cerevisiae* strain TH2-1B [8] or 5288C (*whi5Δ*::*kan^R^*) [36] was used. For certain experiments, the living yeast were labeled with fluorescein isothiocyanate (FITC) as follows. An overnight culture of 5288C was washed twice in PBS and suspended in its original volume in 50 mM Na_2_HPO_4_ (pH 9.4). FITC (Sigma F7250) was added to a final concentration of 1 µg/ml, and the suspension was incubated with shaking for 30 minutes at 37°C. The labeled yeast were washed twice with 50 mM Na_2_HPO_4_ and twice with 17 mM KH_2_PO_4_/Na_2_HPO_4_ buffer, pH 6.0 (PB), before being added to *Dictyostelium* cells. One experiment made use of heat-killed yeast, which had been prepared by boiling for 30 minutes a stock purchased from Sigma (YSC-2); the heat-killed yeast were stored frozen. All yeast cells were washed in PB prior to being added to the *D. discoideum* cells.

For certain experiments, cells were incubated in one-third strength HL5 containing 0.75 mg/ml TRITC-dextran (Sigma, average M_r_ = 66,000) for 3 hours to label all endosomal compartments; yeast were present throughout this incubation. The chamber in which the cells were plated was rinsed to remove the TRITC-dextran immediately before viewing.

### Confocal fluorescence microscopy

For microscopic observation, *D. discoideum* cells in the exponential phase of growth were transferred to a chamber consisting of a plastic ring of 19-mm inner diameter and 4-mm height that had been attached to a cover glass with paraffin. Once the cells had settled, the nutrient medium was replaced with PB. After 30 minutes, yeast were added. A few minutes later (for uptake experiments) or 2 to 6 hours later (for exocytosis experiments), excess yeast were removed, and the cells were overlaid with a thin layer of agarose [37]. The chamber was covered with a second cover glass held in place by silicone grease. Confocal time lapse sequences were captured using an Ultra View ERS-FRET system (Perkin-Elmer LAS) on a TE-2000 microscope equipped with a Plan-Apochromat VC 100x, 1.4 NA objective (Nikon Instruments). Images were acquired at 500-msec or 1-second intervals; GFP and mRFP were excited sequentially with the 488 and 568-nm lines, respectively. The emission was detected through a triple dichroic and a double band pass emission filter onto an EMCCD camera.

For some experiments, confocal images were collected using a Zeiss LSM510 laser scanning confocal microscope equipped with a Plan-Apochromat 63x, 1.4 NA DIC objective. Images were acquired at 3.9-second intervals unless otherwise indicated. S65T-GFP was excited with the 488-nm line of an argon laser with a 505–530-nm filter for emission, and mRFP was excited with the 543-nm line of a HeNe laser, with a 560-nm long pass filter for emission. An HFT UV/488/543/633 beam splitter was used.

## Results

### Delivery of the V-ATPase to new phagosomes

**Figure 1:**
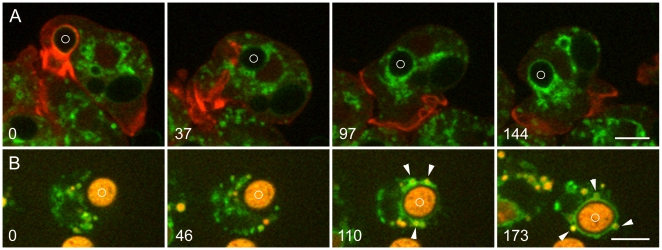
Delivery of the V-ATPase to new phagosomes. A, uptake of living *S. cerevisiae*. Living yeast were added to *Dictyostelium* cells expressing VatM-GFP and mRFP-LimEΔ. At 0 seconds, a phagosome containing a newly ingested yeast (marked with a circle) is surrounded by actin filaments as it moves into the cell. At 37 seconds, the actin filaments have disappeared and VatM-GFP-positive vesicles have clustered around the phagosome; by 97 seconds their numbers have increased. By 144 seconds, transfer of VatM-GFP to the phagosome membrane is evident. Movie S1 contains the complete time series. B, uptake of heat-killed *S. cerevisiae*. *Dictyostelium* cells expressing VatM-GFP were incubated with TRITC-dextran for 4 hours to label all endocytic compartments (endocytic transit time for fluid phase markers being about 1 hour [53]), then heat-killed yeast were added. Some TRITC-dextran penetrated the dead yeast, labeling them as well. At 0 seconds and 46 seconds, a phagocytic cup is extending around a yeast (marked with a circle). In the 110 and 173-second panels, TRITC-dextran content (arrowheads) is evident in several of the VatM-GFP-positive vesicles clustered about the phagosome. Movie S2 contains the complete time series. Perkin-Elmer Ultra View microscope. Bars, 5 µm.

Early stages of phagosome processing in *Dictyostelium* are illustrated in Figure 1. Figure 1A and Movie S1 show uptake of a living yeast by a cell expressing VatM-GFP and mRFP-LimEΔ, a probe for filamentous actin [33], [38]. At time 0, a phagosome whose actin coat identifies it as newly ingested, is propelled away from the site by the formation of an actin tail. After the actin coat has disappeared, numerous small VatM-GFP-positive vesicles surround the phagosome, supplying the V-ATPase to the phagosome membrane.

To investigate the origin of the vesicles that delivered VatM, *Dictyostelium* cells expressing VatM-GFP as the sole fluorescent protein were incubated with TRITC-dextran to label endocytic compartments. Earlier lower-resolution live cell microscopy studies had shown that fusion generates a compartment that contains both the endosomal marker and the phagocytosed particle [8], [29]. Accordingly, the fluid-phase marker revealed that many of the VatM-GFP-positive vesicles that surrounded and fused with a new phagosome are of endosomal origin, confirming that fusion with endolysosomes is an important means of delivering the V-ATPase to the membrane of new phagosomes (Figure 1B and Movie S2). The present study also detected many smaller VatM-GFP-positive vesicles associated with the phagosome that were devoid of visible endosomal content (see Discussion).

### The biosensor GFP-2FYVE, which binds to phosphatidylinositol 3-phosphate, identifies the early endosomal compartment in *Dictyostelium*

**Figure 2:**
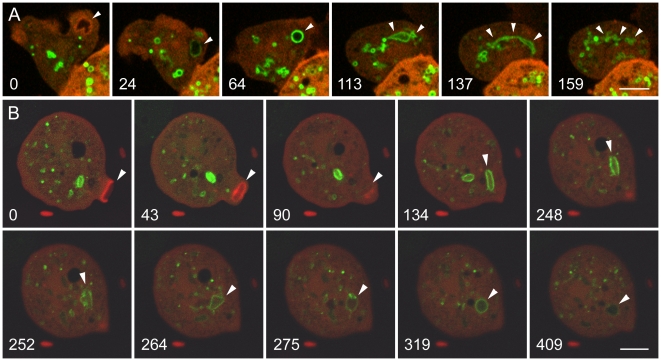
Labeling of early endosomes in *Dictyostelium* cells with GFP-2FYVE, a biosensor for PI(3)P. The cells are expressing GFP-2FYVE and mRFP-LimEΔ. A, a macropinosome (marked by arrowheads in each panel). At 0 seconds, a nascent macropinosome is surrounded by actin filaments. After the actin filaments have disappeared, GFP-2FYVE begins to label the macropinosome, the strongest labeling being seen as the originally amorphous macropinosome assumes a spherical shape about 1 minute after uptake. During the next 2 to 3 minutes, the GFP-2FYVE label gradually fades as the macropinosome becomes increasingly elongated and fragmented. (See Movie S3.) B, phagosomes containing bacteria. The *Dictyostelium* cells were mixed with *E. coli* expressing a low level of a red cytoplasmic marker, and the uptake of two bacteria was tracked. In panel 0, the phagosome containing the first bacterium has already been internalized and has begun binding GFP-2FYVE. A second phagosome is beginning to form (arrowhead), surrounded by actin filaments (red); the completion and internalization of the phagosome is seen in the 43-second and 90-second panels. In subsequent panels, labeling by GFP-2FYVE reveals expansion and morphological changes in the phagosome, including tubular extensions in the 264-second panel. During this period, the GFP-2FYVE labeling fluctuates in intensity, being weaker at 275 seconds than at 319 seconds, and it has largely faded by 409 seconds. (See Movie S4.) A, Perkin-Elmer Ultra View; B, Zeiss LSM510 microscope. Bars, 5 µm.

The delivery of VatM immediately after removal of the actin coat from the phagosome membrane prompted us to define the compartment of the endosomal pathway in which the V-ATPase is acquired. For this purpose we utilized GFP-2FYVE to detect PI(3)P, the phosphoinositide that identifies early endosomes. In *Dictyostelium* cells expressing moderate levels of GFP-2FYVE, phagocytosis and macropinocytosis proceeded normally, as shown in Figure 2. The labeling by GFP-2FYVE of a new macropinosome (Figure 2A) and phagosome (Figure 2B) is shown. The cells are also expressing mRFP-LimEΔ to label the actin filaments that envelop nascent endocytic compartments (arrowheads in both 0 time panels). GFP-2FYVE binds only after the macropinosome or phagosome has sealed and moved into the cell, about one minute after uptake. During the next two minutes, the GFP-2FYVE-labeled macropinosome (A) changes from round to amorphous to elongated to fragmented, corresponding to the tubulo-vesicular sorting stage of the endocytic pathway (Movie S3). Over this interval the GFP-2FYVE binding grows progressively weaker as the PI(3)P content drops; fragmentation and weakened labeling eventually make further tracking impossible.

Figure 2B and Movie S4 show a similar result for a cell that has phagocytosed *E. coli*. The proclivity of early endosomes to undergo fusion and fission is evident in the expanded volume and morphological changes of the phagosome in the 252 and 264-second panels. The GFP-2FYVE signal has largely disappeared by six minutes after uptake. For yeast-containing phagosomes, the duration is somewhat longer and more variable.

The timing and morphology of the GFP-2FYVE binding compartment in *Dictyostelium* resembles PI(3)P signaling in mammalian cells [20], [21], arguing that GFP-2FYVE is an appropriate marker for the early endosomal compartment in *Dictyostelium*. The interval of GFP-2FYVE binding in *Dictyostelium* is the same time period during which the V-ATPase is delivered to new phagosomes [8], and endosomes undergo tubulo-vesicular sorting [39] and become acidified [12].

### Retrieval of the V-ATPase through vesiculation prior to exocytosis

**Figure 3:**
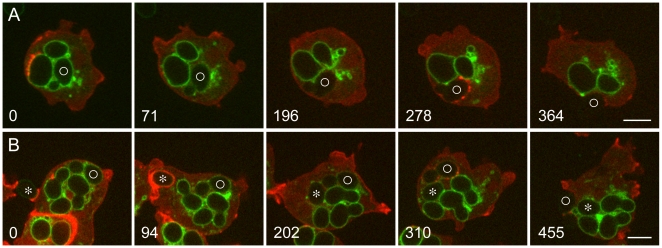
Exocytosis of phagosomes from which the V-ATPase has been removed by vesiculation. A and B, *Dictyostelium* cells expressing VatM-GFP and mRFP-LimEΔ were mixed with living *S. cerevisiae* 2 to 4 hours earlier. In both time series, the cells contain several phagosomes whose membranes are rich in VatM-GFP. A, at 0 seconds, the membrane of a phagosome containing a budded yeast (marked with a circle) is brightly labeled with VatM-GFP. By 71 seconds, the VatM-GFP content of the phagosome membrane has diminished and there is a collection of labeled vesicles nearby. At 196 seconds, VatM-GFP can no longer be detected in the phagosome membrane. At 278 seconds, actin assembly is evident at several points about the phagosome membrane, and by 364 seconds, exocytosis of yeast carcass has been mostly completed. The complete time series is shown in Movie S5. B, at 0 seconds, the upper *Dictyostelium* cell has four yeast-containing phagosomes labeled with VatM-GFP, one of which (marked with a circle) is more weakly labeled than the others. The cell is also beginning to form a phagocytic cup to take up another yeast, marked with an asterisk; at 94 seconds that phagosome, surrounded by actin filaments, has sealed and is moving into the cell. Meanwhile the VatM-GFP content of the phagosome marked with a circle has diminished further, and by 202 seconds VatM-GFP is no longer detectable. At 310 seconds, actin assembly is seen at several points about that phagosome, and its exocytosis is mostly complete at 455 seconds. Meanwhile the membrane of the new phagosome (asterisk) is becoming enriched in VatM-GFP, although that process occurs rather slowly in this cell because so much of the V-ATPase is already in use in the membrane of other phagosomes [8]. The complete time series is shown in Movie S6. Perkin-Elmer Ultra View microscope. Bars, 5 µm.

Seeking to capture exocytosis events, we mixed *Dictyostelium* cells expressing VatM-GFP with living yeast and examined the cells at intervals. Exocytosis of indigestible yeast carcasses could best be observed at 2 to 6 hours after mixing. Such cells were replete with phagosomes whose membranes were rich in VatM-GFP. Incipient exocytosis was characterized by several minutes of vigorous vesiculation at the surface of a VatM-GFP-labeled phagosome. As shown in Figure 3 and Movie S5, this occurred rapidly, over a period of a few minutes. At time 0, the cell in Figure 3A contains three phagosomes whose membranes are rich in VatM-GFP. Over the next three minutes, the membrane of the phagosome marked with a circle loses VatM-GFP, apparently through vesiculation. An indistinct cloud of VatM-GFP-positive vesicles forms at the cytoplasmic face of the phagosome membrane and dissipates, while the phagosome itself moves to and fro. The movement of both the vesicles and the phagosome is consistent with microtubule-based transport. In cells co-labeled with mRFP-LimEΔ, no actin signal is seen. Within two minutes after VatM-GFP has disappeared from the phagosome membrane, actin assembly occurs at several points about the phagosome, possibly positioning it for exocytosis, which occurs a few minutes later. The discontinuous nature of the actin assembly suggests that it is nucleated by effectors associated with microdomains in the phagosome membrane, a possibility consistent with the reported properties of such domains in mammalian cells [40].

A second example of V-ATPase removal prior to exocytosis is shown in Figure 3B and Movie S6; in this case, much of the V-ATPase had already been removed when recording began. During the interval of observation, this cell not only exocytoses a yeast carcass, it also takes up a new yeast particle, underscoring the independent maturation of individual phagosomes.

**Figure 4:**
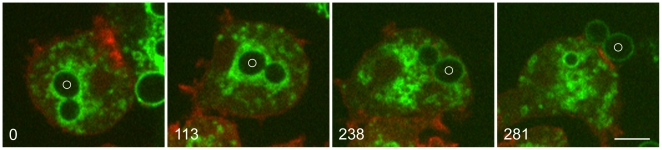
Exocytosis of a FITC-yeast from a phagosome whose membrane has been depleted of VatM-GFP by vesiculation. *Dictyostelium* cells expressing VatM-GFP and DdmCherry-LimEΔ were mixed with FITC-yeast 4 hours earlier. In the 0 and 113-second panels, a phagosome containing a budded yeast is surrounded by a cloud of VatM-GFP-positive vesicles as it undergoes vigorous movement about the cell. This activity extended over a period of about 5 minutes. At 238 seconds, there are no longer vesicles associated with the phagosome membrane, and the FITC-yeast is visible. By 281 seconds, the yeast has been exocytosed with no change in the intensity of the fluorescent signal, indicating that it was not in an acidic compartment at the time of exocytosis. (See Movie S7.) Perkin-Elmer Ultra View microscope. Bar, 5 µm.

We examined the relationship between phagosomal pH and the presence of V-ATPase by feeding cells yeast that had been labeled with FITC, a pH-sensitive fluorophore. FITC fluorescence is quenched at acidic pH, so FITC-yeast in an acidic environment are dim, but brighten when they are neutralized. Figure 4 and Movie S7 show the removal of the V-ATPase from the membrane of a phagosome containing a budded FITC-yeast. In the first two panels, the phagosome membrane is surrounded by a cloud of VatM-GFP-positive vesicles as the V-ATPase is being retrieved; the vigorous dynamics that characterize the retrieval stage are evident in Movie S7. Finally, only the fluorescence of the FITC-yeast shows the position of the phagosome. Note that the intensity of the FITC-yeast fluorescence is similar at 238 seconds (when the yeast is still within the cell) and at 281 seconds (after it has been exocytosed). The interpretation of these data is that VatM-GFP was being removed from the phagosome membrane during the first part of this time series (as in the time series shown in Figure 3 and Movie S5 and S6), and that the fluorescence remaining at 238 seconds was the FITC label on the yeast. The fact that the FITC signal did not brighten upon contact with the extracellular medium argues that the yeast was no longer in an acidic environment at the time of exocytosis and confirms that removal of the V-ATPase correlates with a rise in phagosomal pH.

Removal of the V-ATPase prior to exocytosis of the phagosome appears to be the normal method for retrieval. Although we have recorded only a few examples of the retrieval process using the highly sensitive microscope shown in Figures 3 and 4, we have recorded more than 20 additional examples of the exocytosis of phagosomes devoid of VatM-GFP, often from cells that also contained labeled phagosomes. We have previously published some of these examples [8] [29].

### Retrieval of the V-ATPase upon premature exocytosis

**Figure 5:**
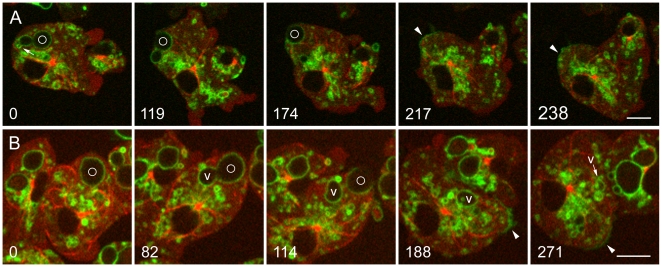
Retrieval of the V-ATPase following premature exocytosis. A and B, *Dictyostelium* cells expressing GFP-α-tubulin and mRFP-LimEΔ were mixed with living *S. cerevisiae* 6 hours earlier. In both time series, a phagosome marked with a circle is exocytosed prematurely, prior to removal of the V-ATPase. A, at 0 seconds, the VatM-GFP-positive phagosome is close to the plasma membrane. A small vacuole brightly labeled with VatM-GFP (arrow) is in the process of budding off from the phagosome membrane. At 119 and 174 seconds, exocytosis is in progress. At 217 seconds, a patch of VatM-GFP is present in the plasma membrane at the site of exocytosis, and a microtubule is lying along the inner surface of the plasma membrane in that area. By 238 seconds, the VatM-GFP signal in plasma membrane is diminishing. The complete time series is shown in Movie S8. B, at 0 seconds, a VatM-GFP-positive phagosome is present near the plasma membrane. At 82 seconds, a large vacuole (V) enriched in VatM-GFP is separating from the phagosome. At 114 seconds, exocytosis of the phagosome is in progress, and at 188 seconds, it has been completed, leaving a bright patch of VatM-GFP in the plasma membrane (arrowhead). This label is much reduced by 271 seconds. (See Supplemental Figure S1 for quantitation.) Meanwhile, the VatM-GFP-positive vacuole (v) has moved about within the cell and undergone a series of morphological changes, including inward budding, which is evident in the 188 and 271-second panels. The complete time series is shown in Movie S9. Perkin-Elmer Ultra View microscope. Bars, 5 µm.

Exocytosis of a phagosome may occur before the V-ATPase has been fully retrieved, a process we call premature exocytosis. This is observed in situations in which cells containing phagosomes with bulky particles are moving through narrow spaces (e.g., squeezing between two other cells). To increase the frequency of premature exocytosis, we used the thin layer of agarose that overlay the cells during our experiments, slightly drying the agarose so that it pressed more strongly on the cells. This technique allowed us repeatedly to record this otherwise uncommon event, two examples of which are shown in Figure 5. The cell in Figure 5A is migrating left to right across the field of view, but its V-ATPase-positive, yeast-containing phagosome is held in place by pressure from the agarose overlay. (This is best appreciated in the full time series, Movie S8.) Thus, although the cell itself is quite motile, its ability to migrate is impeded by the immobilized yeast particle. The outcome of this dilemma is exocytosis of the yeast particle. Some VatM-GFP remains in the phagosome membrane and is transferred to the plasma membrane upon exocytosis. Microtubules make lateral contact with the plasma membrane in that area (217-second frame) and the fluorescent signal begins to diminish (Movie S8), suggesting that the V-ATPase is being carried away from the plasma membrane in the form of vesicles transported along microtubules.

Constraint in the motion of a bulky phagosome appears to be the trigger for premature phagocytosis. We have recorded 14 cases in which phagosomes with VatM-GFP in their membranes were exocytosed. In every case, the phagosome was immobilized, and in 10 of those cases, the cell was motile and attempting to move away from the position of the phagosome. In the remaining cases, the observation period was too brief to make this judgment, or the cell itself was somewhat flattened by the agarose overlay and was not migrating.

Just prior to premature exocytosis, we often observed an abrupt expansion in phagosome volume (11 of the 14 cases described above) followed by separation from the phagosome of one or more large vacuoles whose membranes were rich in VatM-GFP. Such vacuoles were approximately round when they formed and moved away from the phagosome before it was exocytosed. An event of this type is seen in Figure 5B. The vacuole tracks along the cortex for a time, then moves to the cell center. It soon moves outward again, whereupon it elongates into tubular shapes, vesicles appear within its lumen, and it begins to fragment. (The dynamics of the large vacuole and its changing morphology are best appreciated in the accompanying movie, Movie S9.) This sequence of events is remarkably reminiscent of the behavior of an early endosome, a point to which we will return.

Shortly after separation of the vacuole from the phagosome, the phagosome is exocytosed, transferring the remaining phagosomal V-ATPase to the plasma membrane. Note the association of microtubules with that area of the plasma membrane and the diminishing level of VatM-GFP (arrowheads in the last two frames of Figure 5B, and Movie S9). In this case, the recording lasted long enough to allow the reduction of the VatM-GFP signal in the plasma membrane to be measured over time. Approximately two-thirds of the initial VatM-GFP signal disappeared from the plasma membrane over a period of 75 seconds, an interval in which photobleaching was negligible. Details are provided in supplemental Figure S1.

### Phagosomes that undergo premature exocytosis are still acidic and the V-ATPase is still active

**Figure 6:**
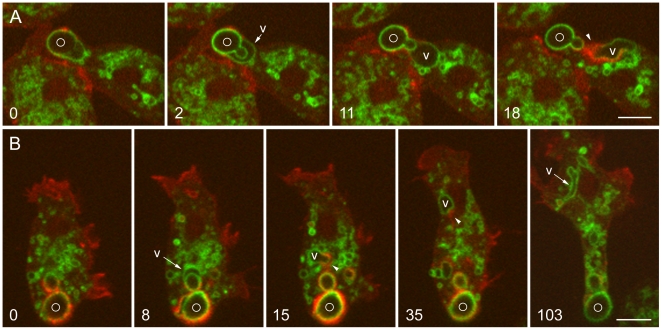
Acidic nature of prematurely exocytosed phagosomes and actin-powered vacuole movement. A and B show *Dictyostelium* cells expressing VatM-GFP and DdmCherry-LimEΔ; the cells were mixed with living FITC-labeled *S. cerevisiae* 5 hours earlier. A, at 0 seconds, VatM-GFP is visible in the phagosome membrane, but the FITC-yeast inside can be seen only faintly. At 2 seconds, a VatM-GFP-positive vacuole (V) is beginning to form and the FITC-yeast has become bright; the yeast bud is now visible. At 11 seconds, the vacuole is separating from the phagosome membrane, and at 18 seconds, it is moving away. New actin assembly detected with DdmCherry-LimEΔ at the rear of the moving vacuole (arrowhead) appears to propel it. The complete time series is shown in Movie S10. B, at 0 seconds, a phagosome containing a budded yeast is faintly green but is more heavily labeled with the red probe for actin filaments. By 8 seconds, the green signal is stronger (appearing yellow where it overlaps the red) as the FITC-yeast has brightened, and a VatM-GFP-positive vacuole (V) has begun to separate from the phagosome. In the 15 and 35-second panels, actin filaments labeled with DdmCherry-LimEΔ can be seen at the rear of the moving vacuole (arrowheads), and by 103 seconds, the vacuole has become an irregular, elongated compartment. The complete time series is shown in Movie S11. Perkin-Elmer Ultra View microscope. Bars, 5 µm.

To test whether phagosomes with the V-ATPase in their membranes are acidic, we sought examples of premature exocytosis by cells that had eaten FITC-yeast. Exocytosis of a FITC-yeast from an acidic phagosome into the higher-pH extracellular buffer should cause the FITC-yeast to fluoresce more brightly. That can be seen to occur in two cases of premature exocytosis shown in Figure 6. In the first panel of Figure 6A, a VatM-GFP-positive phagosome contains a budded yeast marked with a circle, but the yeast itself is not fluorescent, as indicated by the near-invisibility of the constriction at the bud neck. Two seconds later, the FITC-yeast becomes visible as premature exocytosis is initiated; the phagosome expands and a V-ATPase-rich vacuole (V) begins to form. By 18 seconds, the vacuole has separated and is being vigorously propelled away from the site of exocytosis. The probe for filamentous actin expressed in these cells brightly labels the rear of the moving vacuole, revealing that vacuole movement is being powered at least in part by actin assembly (Movie S10).

**Figure 7:**
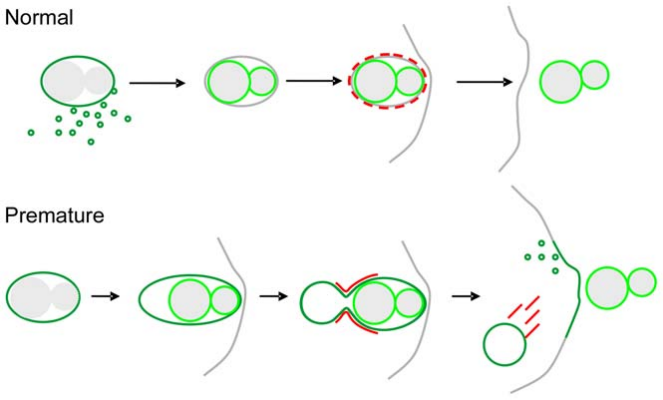
Schematic showing retrieval of the V-ATPase and fluorescence of FITC-yeast during normal and premature exocytosis. Normal exocytosis: Near the end of endocytic transit, the V-ATPase (detected as VatM-GFP) is removed from the phagosome membrane in the form of small vesicles over a period of a few minutes. About two minutes after the V-ATPase has been removed, actin assembles at several points on the phagosome membrane, and exocytosis follows. After removal of the V-ATPase, the FITC-yeast is visible through its own fluorescence, which does not increase further upon exocytosis. Premature exocytosis: The V-ATPase (detected as VatM-GFP) is present in the phagosome membrane. A combination of cell movement and trapping of a bulky phagosome bring the phagosome close to the plasma membrane. An abrupt increase in both the volume of the phagosome and the fluorescence of the FITC-yeast occurs, and a large vacuole whose membrane is rich in the V-ATPase separates from the phagosome and moves away. Actin assembly (shown in red) is instrumental in both the separation of the vacuole and its propulsion through the cytoplasm. Upon exocytosis of the FITC-yeast, the V-ATPase remaining in the phagosome membrane is transferred to the plasma membrane, from which it is soon retrieved. (In the diagram, FITC fluorescence is depicted as a lighter shade of green than GFP fluorescence. However, under our experimental conditions the two signals were not distinguishable.)

Figure 6B and Movie S11 show another case of premature exocytosis involving FITC-yeast. Here the FITC-yeast is faintly visible at time 0, but it brightens in the 8-second frame as the phagosome expands and the beginning of vacuole formation can be seen. In this time series, the vacuole (V) is tracked for a longer period, revealing its movement and the striking morphological changes it undergoes. A schematic representation of the behavior of FITC-yeast and VatM-GFP during normal and premature exocytosis is provided in Figure 7.

**Figure 8:**
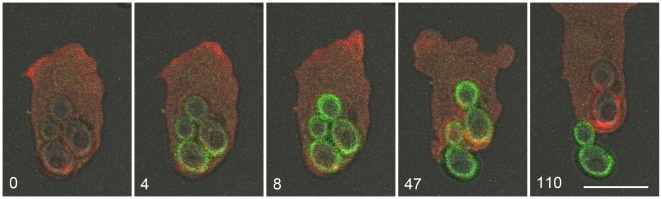
Rapid reacidification of the remaining portion of a multi-particle phagosome following premature exocytosis. This cell is expressing mRFP-LimEΔ, and it was mixed with FITC-yeast 3 hours earlier. At time 0, the yeast are dim, indicating an acidic environment. At 4 seconds, both yeast brighten at the onset of premature exocytosis, indicating contact with the higher pH external medium and demonstrating that they share a single compartment. The phagosome membrane seals behind the first yeast as exocytosis is completed, and the phagosome becomes reacidified, indicating that the V-ATPase remains in active form. Time is shown in seconds. Zeiss LSM510 microscope. Bar, 10 µm.

Evidence that the V-ATPase is still active in the membrane of a phagosome undergoing premature exocytosis is presented in Figure 8. Here, a multi-particle phagosome containing two FITC-yeast underwent premature exocytosis. As is typical for exocytosis of large particles from a multi-particle phagosome, the plasma membrane sealed behind the first particle as it was released, and there was a lag before exocytosis of the second particle [41]. This circumstance provided a clear demonstration that the V-ATPase was still present and active in the membrane of the second phagosome, because FITC fluorescence of the second yeast was rapidly quenched as that compartment became re-acidified.

### Vacuoles formed during premature exocytosis are associated with myosin-IB as well as actin

**Figure 9:**
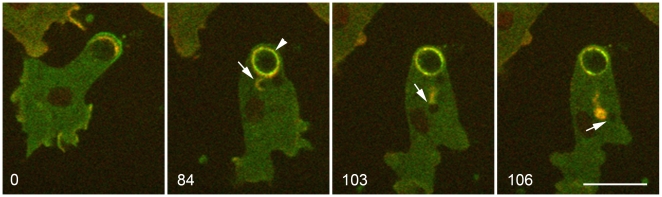
Recruitment of myosin-IB at the onset of premature exocytosis. This cell is expressing GFP-MyoB and mRFP-LimEΔ; it was mixed with unlabeled yeast two hours earlier. The cell is initially migrating (0 time), but the bulky phagosome soon becomes immobilized. At 84 seconds, the immobilized phagosome has brightened (arrowhead), indicating binding of GFP-MyoB, and a vacuole (tailed arrow) is just separating from the phagosome. The vacuole travels across the cell, labeled with GFP-MyoB and trailed by an actin tail (103 and 106 seconds). Perkin-Elmer Ultra View microscope. Bar, 10 µm.

Cells expressing fluorescently-tagged myosin-IB (GFP-MyoB) revealed that, together with actin, myosin-IB is involved in premature exocytosis. We showed earlier that if a phagosome makes contact with the cortical actin layer, GFP-MyoB becomes transiently enriched at the plasma membrane at the point of contact, and there follows a brief spurt of actin-mediated rocketing that moves the phagosome away from the cortex [42]. In the first panel of Figure 9, such an event is occurring at the rear of a phagosome, moving the phagosome along as the cell migrates. About one minute later, this phagosome becomes immobilized, initiating the sequence of events that accompany premature exocytosis. In the 84-second panel, a vacuole has just separated from the phagosome (arrowhead). Just preceding that separation, the entire phagosome membrane becomes brightly labeled with GFP-MyoB, indicating a change in the binding properties of the phagosome membrane at the onset of premature exocytosis. In the 103 and 106-second panels, GFP-MyoB is also present at the membrane of the vacuole as it moves across the cell ahead of an actin tail, suggesting that myosin-IB may contribute to vacuole movement.

### Source of phagosome expansion and vacuole content

**Figure 10:**
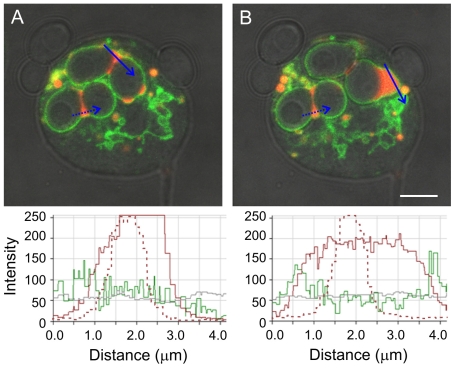
Increase in phagosome volume and dilution of fluid phase marker prior to premature exocytosis. This cell was incubated in the presence of both TRITC-dextran and yeast for 3 hours, then rinsed with buffer, covered with a layer of agarose, and observed immediately. This time series (Movie S12) was captured 4 minutes after observation began, so all endosomal compartments except new macropinosomes should be filled with TRITC-dextran. The cell has two V-ATPase-positive phagosomes, each containing two yeast particles and some TRITC-dextran. The phagosome at the upper right expands, a vacuole separates from it, and the two yeast are sequentially exocytosed (although only the first exocytosis is shown). The graphics below the images display the intensity per pixel in the red, green, and brightfield channels along the blue line in each image, as determined by the Zeiss AIM software. The maximum possible intensity value is 255 for these 8-bit images. Although the intensity of the TRITC-dextran (red) signal has saturated the detector in the first image (A), it is clear that the red pixel intensity diminishes in the second image (B) as the phagosome expands, indicating that at least part of the new fluid has come from an unlabeled source. The two small endosomes in the process of fusing at the bottom of the phagosome in A also had saturated peak pixel intensities (not shown); these were incorporated when the phagosome expanded, contributing some additional TRITC-dextran. As a control for photobleaching, a second phagosome with TRITC-dextran content at the bud neck was also scanned (dashed arrows); its TRITC-dextran signal is shown by the dashed lines on the plot. There was no change in pixel intensity between the two time points for the TRITC-dextran in this phagosome. Movie S12 shows the expansion of the phagosome and the dynamic behavior of the vacuole that separates from the phagosome, including the formation of intraluminal vesicles. In the bottom right quadrant of this cell, one can also see the action of the contractile vacuole complex, an osmoregulatory organelle found on the cytoplasmic surface of the substratum-attached plasma membrane; contractile vacuole membranes are also rich in the V-ATPase. Additional time points and a second example are shown in Supplemental Figures S2 and S3. Zeiss LSM 510 microsope.

In premature exocytosis, a sudden expansion of phagosome volume commonly precedes the release of V-ATPase-rich vacuoles from the phagosome. We investigated the source of this volume increase using cells that were maintained in the presence of TRITC-dextran for three hours while they were incubated with yeast, in order to label all endosomal compartments. The cells were rinsed with buffer immediately before they were observed. We were thus able to ask whether the volume increase that preceded vacuole release arose from fusion with TRITC-labeled endosomes or with an unlabeled source. The TRITC-dextran content of the phagosome was diluted by the fusion event, indicating that the source of the fluid influx was at least partly unlabeled (Figure 10 and Movie S12). (Additional details and a second example are provided in supplemental Figures S2 and S3.) The likeliest source of the unlabeled fluid appears to be the extracellular medium (see Discussion). The fact that FITC-yeast suddenly became bright, indicating an abrupt pH increase as volume expansion and vacuole formation were occurring (Figure 6), is consistent with this interpretation.

Another potential source of additional membrane and unlabeled fluid is the contractile vacuole complex, an osmoregulatory compartment that is separate from the endosomal system and does not acquire dextran applied to the external medium [43]. To test this possibility, we sought an example of premature exocytosis in cells expressing dajumin-GFP, a marker specific for the contractile vacuole system [43]. The expanded phagosome and released vacuole were not labeled with dajumin-GFP, arguing against a role for the contractile vacuole system in this process (supplemental Figure S4).

### The V-ATPase-rich vacuole released in premature exocytosis acquires the properties of an early endosome

The identity of successive compartments along the endocytic pathway is primarily specified by their phosphoinositide composition [21] [18]. Biosensors for phosphoinositides allow identification of the first two stages of endocytic transit in living cells. PHcrac-GFP binds to PI(3,4,5)P3 and PI(3,4)P2, which are enriched on the membrane of nascent and just-sealed endosomes [44] [29], and 2FYVE-GFP binds to PI(3)P, which is enriched on the membrane of early or sorting endosomes, as illustrated earlier in this report. Neither of these biosensors binds to mature endosomes, which are thought to be enriched in PI(3,5)P2 and lyso*bis*phosphatidic acid [21]. As noted earlier, the dynamics and changing morphology of the V-ATPase-rich vacuoles released from phagosomes during premature exocytosis are reminiscent of early endosomes. We therefore examined whether the vacuoles formed upon premature exocytosis are recycled to the early endosome stage.

**Figure 11:**
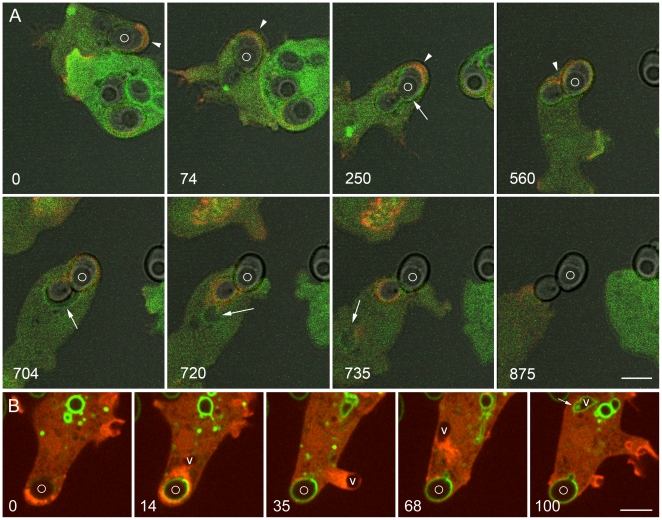
Changes in the phosphoinositide composition of the phagosome and vacuole in premature exocytosis. A, these *Dictyostelium* cells, expressing PHcrac-GFP and mRFP-LimEΔ, were mixed with unlabeled yeast one and a half hours earlier. The upper, more weakly labeled cell is migrating. Arrowheads indicate the direction of cell movement or attempted movement in the first four panels. A phagosome containing a budded phagosome (marked with a circle) is initially pushed along by actin assembly (red), but the phagosome becomes increasingly constrained and presently ceases to move. At the same time, PHcrac-GFP begins to bind to the phagosome membrane (tailed arrow in 250-second panel), indicating the presence of PI(3,4,5)P3 and/or PI(3,4)P2. This biosensor also labels the expanded phagosome (704 seconds) and the vacuole that separates from it (720 and 735 seconds) (tailed arrows). Meanwhile, the phagosome is exocytosed. B, this *Dictyostelium* cell, expressing GFP-2FYVE and mRFP-LimEΔ, was mixed with FITC-yeast 4 hours earlier. At 0 seconds it contains a phagosome in which the FITC-yeast is barely visible, indicating that the phagosome is acidic. At 14 seconds the FITC-yeast has brightened, indicating a rise in pH within the phagosome, and a vacuole (V) has separated from the phagosome. The vacuole moves rapidly away from the phagosome at the head of a trail of actin filaments, creating a protrusion in plasma membrane at 35 seconds, then rebounding to the cytoplasm at 68 seconds. By 100 seconds the vacuole is binding GFP-2FYVE and has assumed the elongated morphology of an early endosome. Meanwhile the FITC-yeast has been exocytosed. Movie S13 shows the complete time series. A, Zeiss LSM510 microscope; B, Perkin-Elmer Ultra View microscope. Bars, 5 µm.

Figure 11A shows two cells expressing PHcrac-GFP and mRFP-LimEΔ that were mixed with budded yeast one and a half hours earlier. The upper cell is migrating, initially from right to left (panel 0), then down around the other cell (74 seconds), then diagonally to the left (250 seconds). A yeast-containing phagosome at the rear of the cell is being boosted along by actin assembly each time it contacts the cortex. However, by 250 seconds the phagosome has ceased to move. The cell tries to migrate downward (560 seconds), then diagonally again (704 seconds), but the phagosome remains stationary. Presumptive premature exocytosis follows, signaled by phagosome expansion (704 seconds), vacuole release (720 seconds), and exocytosis of the phagosome. Note that as phagosome movement slows and stops, the phagosome membrane becomes labeled with PHcrac-GFP, and this biosensor for new endosomes also labels the expanded phagosome and the vacuole that separates from it (tailed arrows).

We next sought examples of premature exocytosis in cells that had eaten FITC-yeast and were expressing GFP-2FYVE and mRFP-LimEΔ. Figure 11B and Movie S13 show such an event. Although this cell was not expressing VatM-GFP, we can infer the presence of the V-ATPase in the phagosome membrane from the brightening of the FITC-yeast when it contacted the extracellular medium, indicating that the phagosome was still acidic up to the time of fusion with the plasma membrane. Powerful actin-based propulsion of a large vacuole away from the phagosome just prior to exocytosis is seen. Note that the vacuole is propelled so strongly that it creates a protrusion (35-second panel). However, it does not fuse with the plasma membrane. Instead, it rebounds into the cytoplasm, where less than 2 minutes after its formation, the vacuole changes it shape and becomes capable of binding GFP-2FYVE. Thus, the vacuole membrane now carries PI(3)P, the phosphoinositide that specifies the binding and fusion capabilities of early endosomes [18]. It appears therefore that such vacuoles provide a rapid and direct means of recycling the V-ATPase to the beginning of the endocytic pathway.

## Discussion

The present study has visualized trafficking of the V-ATPase in both early and late stages of the endocytic pathway. After the actin filaments that shaped the phagocytic cup and propelled the phagosome away from the cortex had disappeared, V-ATPase-rich vesicles clustered around the new phagosome. Fluid phase content detected within the lumen of several of these vesicles indicated that they were of endosomal origin [8]. There were also many small vesicles free of detectable endosomal content. These too may be of endosomal origin, but derived from a recycling step in which membrane-enriched vesicles separate from compartments enriched in endosomal content [45]. Within three minutes, the membrane of the new phagosome grew brightly labeled with VatM-GFP. Similarly, it was recently reported that nascent phagosomes in mouse macrophages receive the a3 subunit of the V-ATPase from tubular extensions of lysosomes soon after losing actin filaments, and that genetic loss of the a3 subunit results in severe impairment of phagosome acidification [24].

The novel contribution of the present study is the visualization of multiple routes for retrieval of the V-ATPase from phagosome membranes. In normal retrieval, small vesicles rich in VatM-GFP were carried away from the phagosome membrane over a period of several minutes shortly before exocytosis of the neutralized phagosome. The vigorous movement of both the phagosome and the vesicles during this period was suggestive of microtubule-based transport. We were unable to track the fate of individual vesicles owing to their small size and rapid motion. However, it is appealing to speculate that they may form part of the complement of small vesicles that surround and fuse with a new phagosome. After VatM-GFP had been completely removed, strong actin assembly occurred at several points about the phagosome membrane, apparently positioning the phagosome for exocytosis, which followed a few minutes later. This result is consistent with biochemical analysis of isolated phagosomes [5], with reports that coats of actin or actin-binding proteins can be found on late neutral endosomes, but not on acidic endosomes [10], [46], [47], and with the finding that a GFP-actin binding domain probe associates with endosomal vesicles shortly before exocytosis [48].

Our experiments using FITC-yeast showed a correlation between removal of the V-ATPase from the phagosome membrane and a rise in luminal pH. It has been demonstrated in macrophages that inactivation of the V-ATPase by inhibitors results in an elevation in luminal pH owing to a passive proton leak across the phagosome membrane [49], [50]. Thus, removal of the V-ATPase is probably sufficient to account for the rise in pH commonly observed for late endosomes in *Dictyostelium* cells prior to exocytosis [51], [52], [53]. Earlier studies of endocytic transit in populations of *Dictyostelium* cells led to models in which late endosomes filled with fluid phase markers existed for extended periods in a neutralized state prior to exocytosis [46], [51]. However, for individual phagosomes, we find that removal of the V-ATPase, a rise in endosomal pH, and exocytosis all occur within a period of about 10 minutes, with about half of that time being devoted to removal of the V-ATPase from the phagosome membrane.

At present there is no information regarding what triggers the removal of the V-ATPase from the phagosome membrane, although increasing acidity within the maturing phagosome may play a role. The V-ATPase itself can act as a pH sensor, interacting with small GTPases in an acidification-dependent manner to modulate vesicular trafficking [3], [54], [55]. It has therefore been suggested that the V-ATPase may regulate its own trafficking [31].

An unexpected discovery was that in confined spaces, the phagocytes tended to exocytose a large particle prior to complete removal of the V-ATPase from the phagosome membrane. Premature exocytosis commonly occurred when a cell's mobility in a confined space was limited by a bulky phagosome. In migrating cells, a phagosome is normally kept away from the cell cortex by a brief pulse of actin assembly that is induced each time the phagosome approaches the cortex [42]. When phagosome movement is constrained, this mechanism fails, and exocytosis soon follows. In the present study, constraint was applied in the form of pressure from a thin sheet of agarose. However, we suggest that *Dictyostelium* amoebae may also encounter narrow passageways as they crawl among soil particles in their natural environment, and this might also occur for mammalian phagocytes migrating within the intercellular spaces of a tissue.

In premature exocytosis, some VatM-GFP from the phagosome membrane was transferred to the plasma membrane. This signal declined rapidly, apparently through the internalization of small vesicles. The rapid rate of removal suggests that a specific and efficient retrieval mechanism is employed. An earlier electron microscopy study of quick-frozen *Dictyostelium* cells found that V-ATPase complexes mislocalized to the plasma membrane rapidly became surrounded by clathrin lattices [56], suggesting that the V-ATPase may be removed from the plasma membrane in a clathrin-dependent manner. This possibility remains to be explored.

When an immobilized phagosome is pressed against the plasma membrane as a cell attempts to migrate, the phosphoinositide composition of the phagosome membrane changes; it becomes capable of binding PHcrac-GFP, a biosensor for PI(3,4,5)P3 and PI(3,4)P2, phosphoinositides that are normally restricted to nascent and just-sealed endosomes [44]. Possibly, the close proximity of the phagosome to the plasma membrane brings it into contact with a resident kinase that effects this conversion. A few minutes later, the phagosome expands with an influx of fluid that appears to come from the extracellular environment. This may be due to an osmotically-driven influx of buffer as the acidic phagosome first becomes connected to the extracellular space. The result is an abrupt increase in phagosome volume, diluting the luminal contents and elevating its pH. Often, shortly after this volume increase, a V-ATPase-rich vacuole is seen to separate from the phagosome and rocket away with an elongating actin tail at its back. Myosin-IB may be instrumental in this process, since GFP-MyoB is recruited to the phagosome just before the vacuole forms and is associated with the moving vacuole. The V-ATPase-rich vacuole assumes the elongated morphology and dynamic behavior of early endosomes, an identity confirmed by the binding of GFP-2FYVE to such a vacuole less than two minutes after its formation. Thus, in premature exocytosis, a significant fraction of the V-ATPase present in the phagosome membrane is recycled directly back to the early endosomal compartment, where it is available for fusion with newly formed endosomes and phagosomes.

In conclusion, our live cell microscopy studies have revealed that *Dictyostelium* cells are capable of utilizing three different routes for retrieving the V-ATPase from phagosome membranes. Commonly, V-ATPase retrieval occurs through vesiculation shortly before exocytosis of a neutralized phagosome. When constraint of a bulky phagosome leads to premature exocytosis, the separation of a large vacuole prior to exocytosis allows recovery of a portion of the V-ATPase, and the remainder is rapidly retrieved from the plasma membrane. This versatility presumably accounts for our earlier finding [30] that the enzyme is efficiently recycled in spite of the high-throughput endocytic activities of this professional phagocyte. The discovery of a retrieval route in which a large vacuole splits off from the phagosome prior to exocytosis enabled us to record the retrograde pathway from the final step of exocytosis back to an early endosome.

## Supporting Information

Figure S1 Removal of VatM-GFP from the plasma membrane following premature exocytosis. Frames from the time series shown in Figure 5B and Movie S9 were analyzed to quantify the rate of disappearance of VatM-GFP from the plasma membrane following premature exocytosis. Arrowheads mark the patch of plasma membrane labeled with VatM-GFP; frames separated by 25-second intervals were analyzed. The frames were exported to Image J in 8-bit format and background-subtracted frame-by-frame. Using the free-hand tool, the perimeter of the cell was outlined. For each frame, the intensity of green fluorescence (VatM-GFP) was averaged over a 3-pixel profile width along the 15-µm segment marked by the yellow line. Bleaching was measured for the entire cell over the 75-second period and found to be negligible (∼5%). However, more than two-thirds of the VatM-GFP signal was removed during this 75-second interval, as shown in the graphical display. Perkin-Elmer Ultra View microscope.
(2.06 MB TIF)

Figure S2 Increase in phagosome volume and dilution of fluid phase marker prior to premature exocytosis. (A) The cell was incubated with TRITC-dextran and yeast for three hours, then the medium was replaced with buffer, the cells were covered with a thin layer of agarose that was slightly dried to induce premature exocytosis, and the sample was viewed at once. All endosomal compartments except new macropinosomes were expected to contain TRITC-dextran. (This is the same experiment shown in Figure 10 but including additional time points; see that legend for further details.) The red pixel intensity drops when the upper multi-particle phagosome expands (43 seconds), indicating an influx of unlabeled fluid. A vacuole separates from the phagosome and moves about the cell (91 to 477 seconds), and the first yeast in the multiparticle phagosome is exocytosed (370 seconds). Sorting and recycling concentrate the TRITC-dextran in the vacuole, increasing the pixel intensity once again (370 and 477 seconds); internal vesicles can be seen within the vacuole at 477 seconds. Zeiss LSM510 microscope.
(3.35 MB TIF)

Figure S3 Increase in phagosome volume and dilution of fluid phase marker prior to premature exocytosis. (B) This sample was prepared as described in (A) except that the cells were left in buffer for 30 minutes prior to viewing. Thus, late but not early endosomes were expected to contain TRITC-dextran. The results were similar to those in the first experiment, namely, expansion of the phagosome and dilution of the TRITC-dextran (20 seconds), separation of a vacuole (43 seconds), exocytosis of the yeast (79 and 173 seconds), and an increase in TRITC-dextran concentration as the volume of the vacuole is reduced during sorting (173 and 264 seconds). Zeiss LSM510 microscope.
(3.11 MB TIF)

Figure S4 Phagosome expansion and vacuole separation in a cell expressing the contractile vacuole marker dajumin-GFP. The cells were expressing dajumin-GFP and mRFP-LimEΔ; they were mixed with yeast two hours earlier. The cells were covered with a thin layer of agarose that was dried slightly to induce premature exocytosis. A vacuole (arrowhead) separates from the phagosome (39 seconds) and moves away with a tail of actin filaments (53 and 79 seconds). Meanwhile, the phagosome is exocytosed (53 and 79 seconds). No dajumin-GFP label is incorporated into the membrane of the phagosome or the vacuole, arguing that the contractile vacuole system is not the source of the added membrane and fluid. Zeiss LSM510 microscope. Bar, 5 µm.
(2.05 MB TIF)

Movie S1 Delivery of the V-ATPase to a new phagosome. The cell is expressing VatM-GFP and mRFP-LimEΔ. It has just phagocytosed a living yeast cell.
(8.16 MB MOV)

Movie S2 Delivery of the V-ATPase to a new phagosome. The cell is expressing VatM-GFP and has been incubated with TRITC-dextran to label endosomes. The uptake of a heat-killed yeast is shown.
(2.03 MB MOV)

Movie S3 Labeling of a macropinosome with GFP-2FYVE, a biosensor for PI(3)P. The cell is expressing GFP-2FYVE and mRFP-LimEΔ. The nascent macropinosome is initially labeled with the actin marker. Actin disappears and 2FYVE-GFP binding begins after about one minute. It persists for another 2 or 3 minutes as the macropinosome elongates and fragments, behavior consistent with the early or sorting endosome stage.
(1.23 MB MOV)

Movie S4 Labeling by GFP-2FYVE of phagosomes containing bacteria. The cell is expressing GFP-2FYVE and mRFP-LimE and is eating bacteria. New phagosomes are labeled first by the actin marker and then by GFP-2FYVE starting about one minute later and persisting for several minutes. Fusion and fission events characteristic of early phagosomes can be seen during the period of 2FYVE-GFP labeling.
(4.84 MB MOV)

Movie S5 Removal of the V-ATPase from the membrane of a yeast-containing phagosome, followed by actin assembly and exocytosis. The cell is expressing VatM-GFP and mRFP-LimEΔ.
(8.65 MB MOV)

Movie S6 Removal of the V-ATPase from the membrane of a yeast-containing phagosome, followed by actin assembly and exocytosis. Over the same interval, the cell takes up a new yeast. The cell is expressing VatM-GFP and mRFP-LimEΔ.
(8.46 MB MOV)

Movie S7 Exocytosis of a FITC-yeast from a phagosome whose membrane has been depleted of VatM-GFP prior to exocytosis. The cell is expressing VatM-GFP and DdmCherry-LimEΔ. The yeast does not grow brighter upon contact with the extracellular medium.
(9.33 MB MOV)

Movie S8 Premature exocytosis of a yeast-containing phagosome and initial stage of the retrieval of VatM-GFP from the plasma membrane. The cell is expressing VatM-GFP and mRFP-α-tubulin.
(7.22 MB MOV)

Movie S9 Separation of a large V-ATPase-rich vacuole from a yeast-containing phagosome prior to premature exocytosis. The cell is expressing VatM-GFP and mRFP-α-tubulin.
(8.50 MB MOV)

Movie S10 Acidic nature of a prematurely exocytosed phagosome, also showing actin-powered vacuole movement. The cell is expressing VatM-GFP and DdmCherry-LimEΔ. It has eaten a FITC-labeled yeast that is barely visible in the phagosome but brightens upon contact with the higher pH extracellular medium, indicating that the phagosome lumen was acidic.
(3.02 MB MOV)

Movie S11 Premature exocytosis of a phagosome containing a FITC-yeast and dynamics of the vacuole that separates prior to exocytosis. The cell is expressing VatM-GFP and DdmCherry-LimEΔ.
(3.37 MB MOV)

Movie S12 Increase in phagosome volume and dilution of fluid phase marker prior to premature exocytosis. The cell is expressing VatM-GFP and was incubated for 3 hours with TRITC-dextran, then shifted to unlabeled buffer and observed.
(9.60 MB MOV)

Movie S13 Acquisition of GFP-2FYVE label by a vacuole that has separated from a phagosome just prior to premature exocytosis. The cell is expressing GFP-2FYVE and mRFP-LimEΔ; the phagosome contains a FITC-yeast.
(3.86 MB MOV)
