# Supplementary figures and images for: Retrieval of the Vacuolar H+-ATPase from Phagosomes Revealed by Live Cell Imaging
Source: PLoS One. 2010 Jan 5;5(1):e8585. doi: 10.1371/journal.pone.0008585 (PMC2796722; doi:10.1371/journal.pone.0008585)

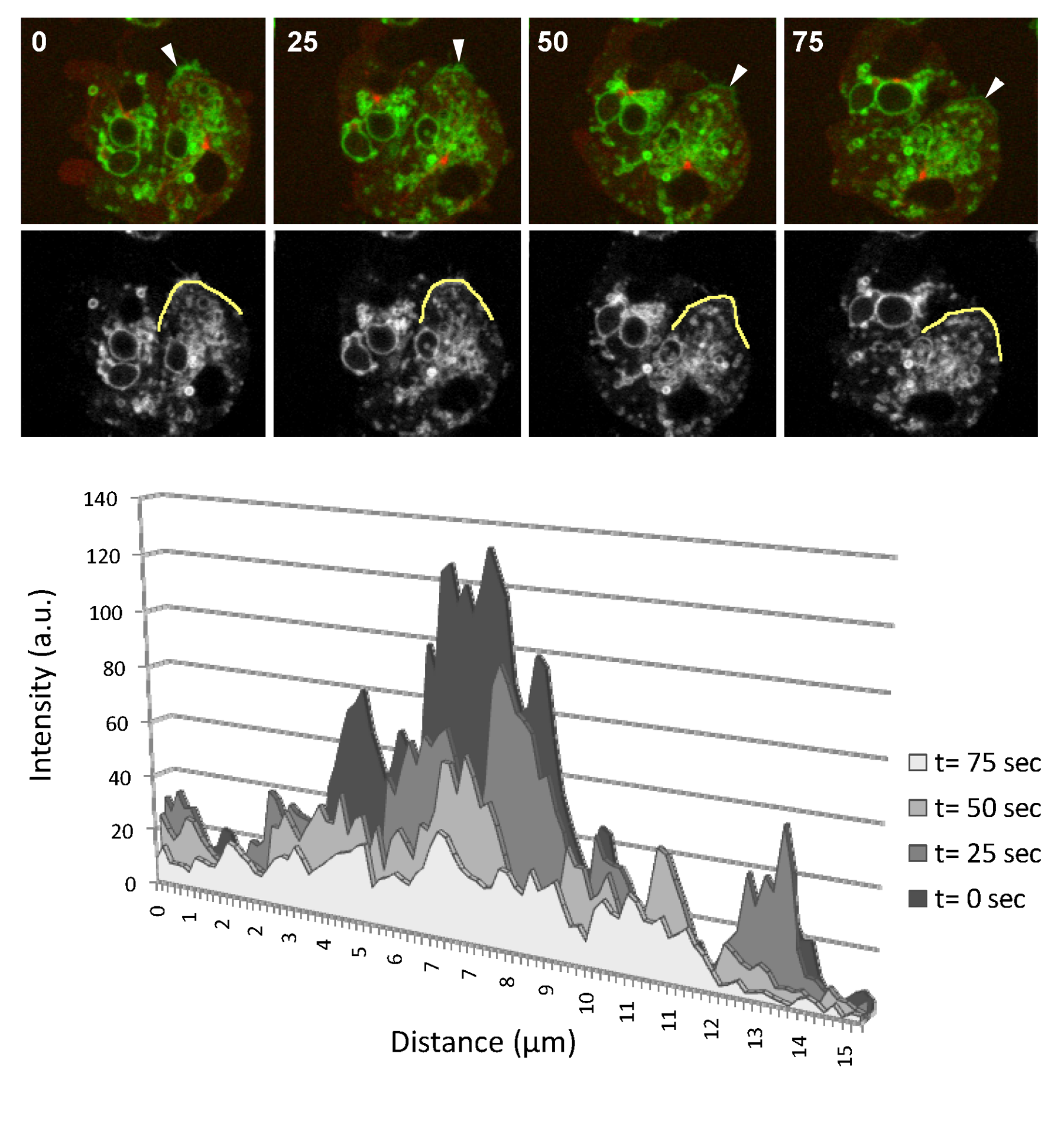

Supplement: Figure S1 — Removal of VatM-GFP from the plasma membrane following premature exocytosis. Frames from the time series shown in Figure 5B and Movie S9 were analyzed to quantify the rate of disappearance of VatM-GFP from the plasma membrane following premature exocytosis. Arrowheads mark the patch of plasma membrane labeled with VatM-GFP; frames separated by 25-second intervals were analyzed. The frames were exported to Image J in 8-bit format and background-subtracted frame-by-frame. Using the free-hand tool, the perimeter of the cell was outlined. For each frame, the intensity of green fluorescence (VatM-GFP) was averaged over a 3-pixel profile width along the 15-µm segment marked by the yellow line. Bleaching was measured for the entire cell over the 75-second period and found to be negligible (∼5%). However, more than two-thirds of the VatM-GFP signal was removed during this 75-second interval, as shown in the graphical display. Perkin-Elmer Ultra View microscope. (2.06 MB TIF) [file pone.0008585.s001.tif]

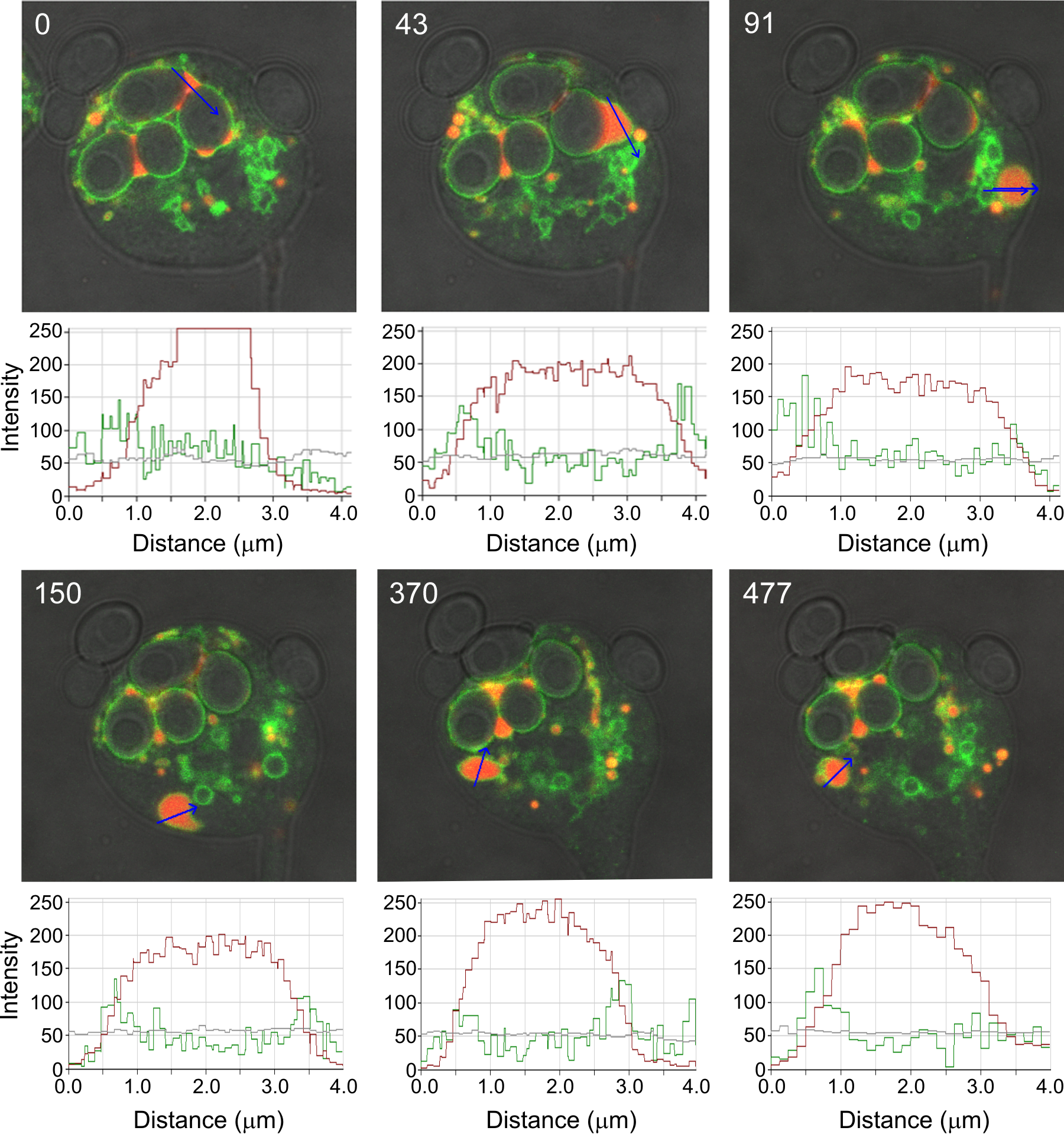

Supplement: Figure S2 — Increase in phagosome volume and dilution of fluid phase marker prior to premature exocytosis. (A) The cell was incubated with TRITC-dextran and yeast for three hours, then the medium was replaced with buffer, the cells were covered with a thin layer of agarose that was slightly dried to induce premature exocytosis, and the sample was viewed at once. All endosomal compartments except new macropinosomes were expected to contain TRITC-dextran. (This is the same experiment shown in Figure 10 but including additional time points; see that legend for further details.) The red pixel intensity drops when the upper multi-particle phagosome expands (43 seconds), indicating an influx of unlabeled fluid. A vacuole separates from the phagosome and moves about the cell (91 to 477 seconds), and the first yeast in the multiparticle phagosome is exocytosed (370 seconds). Sorting and recycling concentrate the TRITC-dextran in the vacuole, increasing the pixel intensity once again (370 and 477 seconds); internal vesicles can be seen within the vacuole at 477 seconds. Zeiss LSM510 microscope. (3.35 MB TIF) [file pone.0008585.s002.tif]

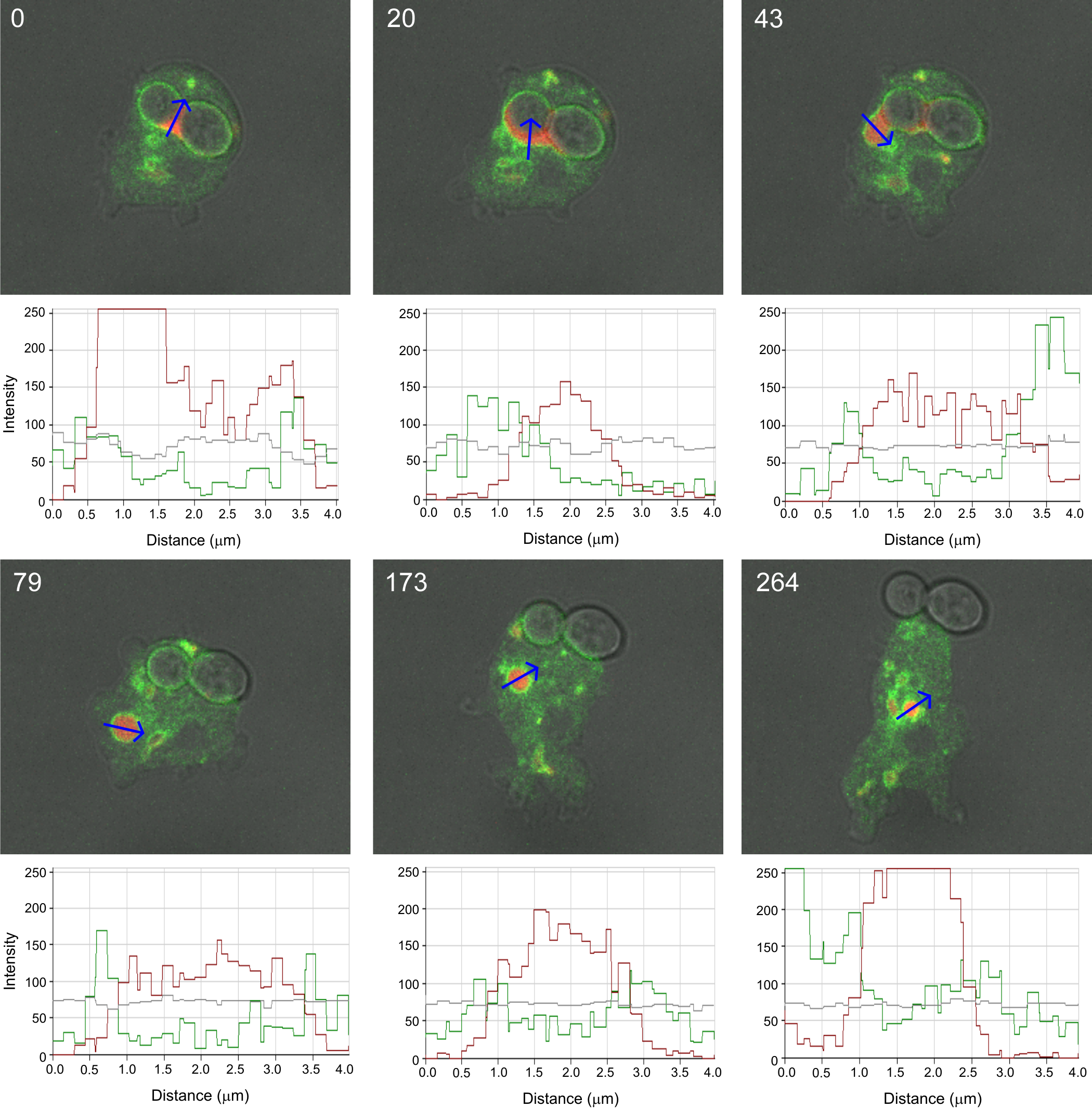

Supplement: Figure S3 — Increase in phagosome volume and dilution of fluid phase marker prior to premature exocytosis. (B) This sample was prepared as described in (A) except that the cells were left in buffer for 30 minutes prior to viewing. Thus, late but not early endosomes were expected to contain TRITC-dextran. The results were similar to those in the first experiment, namely, expansion of the phagosome and dilution of the TRITC-dextran (20 seconds), separation of a vacuole (43 seconds), exocytosis of the yeast (79 and 173 seconds), and an increase in TRITC-dextran concentration as the volume of the vacuole is reduced during sorting (173 and 264 seconds). Zeiss LSM510 microscope. (3.11 MB TIF) [file pone.0008585.s003.tif]

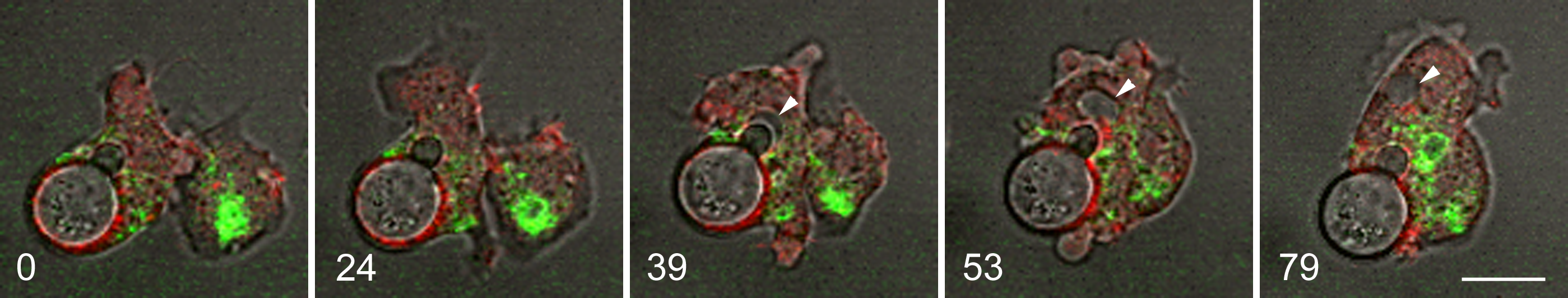

Supplement: Figure S4 — Phagosome expansion and vacuole separation in a cell expressing the contractile vacuole marker dajumin-GFP. The cells were expressing dajumin-GFP and mRFP-LimEΔ; they were mixed with yeast two hours earlier. The cells were covered with a thin layer of agarose that was dried slightly to induce premature exocytosis. A vacuole (arrowhead) separates from the phagosome (39 seconds) and moves away with a tail of actin filaments (53 and 79 seconds). Meanwhile, the phagosome is exocytosed (53 and 79 seconds). No dajumin-GFP label is incorporated into the membrane of the phagosome or the vacuole, arguing that the contractile vacuole system is not the source of the added membrane and fluid. Zeiss LSM510 microscope. Bar, 5 µm. (2.05 MB TIF) [file pone.0008585.s004.tif]
